# BayesCNV: A Bayesian Hierarchical Model for Sensitive and Specific Copy Number Estimation in Cell Free DNA

**DOI:** 10.3390/diagnostics16020280

**Published:** 2026-01-16

**Authors:** Austin Talbot, Alex Kotlar, Lavanya Rishishwar, Andrew Conley, Mengyao Zhao, Nachen Yang, Michael Liu, Zhaohui Wang, Sean Polvino, Yue Ke

**Affiliations:** Pillar Biosciences Inc., Natick, MA 01760, USA; kotlara@pillarbiosci.com (A.K.); rishishwarl@pillarbiosci.com (L.R.); conleya@pillarbiosci.com (A.C.); zhaom@pillarbiosci.com (M.Z.); yangn@pillarbiosci.com (N.Y.); lium@pillarbiosci.com (M.L.); wangzh@pillarbiosci.com (Z.W.); polvinos@pillarbiosci.com (S.P.)

**Keywords:** Bayesian hierarchical model, copy number variation, liquid biopsy, next-generation sequencing, probabilistic machine learning, targeted sequencing, thermodynamic integration

## Abstract

**Background/Objectives**: Detecting copy number variations (CNVs) from next-generation sequencing (NGS) is challenging, particularly in targeted sequencing panels, especially for cell-free DNA (cfDNA), where the signal is weak and noise is high. **Methods**: We present BayesCNV, a Bayesian hierarchical model for gene-level copy ratio estimation from targeted amplicon read depths compared to a CNV-neutral reference sample. The model provides posterior uncertainty for each gene and supports interpretable calling based on effect size and posterior confidence. The model also provides a principled quality-control strategy based on the marginal log likelihood of each sample, with low values indicating low confidence in the calls. BayesCNV uses thermodynamic integration, a technique to reliably estimate this quantity. We benchmark our method against two publicly available CNV callers using Seracare^®^ reference samples with known CNVs on the OncoReveal^®^ Core Lbx panel. **Results**: Our method achieves a sensitivity of 0.87 and specificity of 0.996, dramatically outperforming two competitor methods, IonCopy and DeviCNV. In a separate FFPE dataset using the OncoReveal^®^ Essential Lbx panel, we show that the marginal log likelihood cleanly separates, degraded from high-quality samples, even when conventional sequencing QC metrics do not. **Conclusions**: BayesCNV provides accurate and interpretable gene-level CNV estimates and uncertainty quantification, along with an evidence-based quality control metric that improves robustness in targeted cfDNA workflows.

## 1. Introduction

Copy number variations (CNVs) are genomic aberrations caused by an excess or deficit in the number of copies of a genomic region or gene [1] relative to the typical diploid state. These aberrations are involved in the onset and progression of various diseases, including cancer [2], heart disease [3], and thalassemia [4]. Accurate identification of CNVs from next-generation sequencing (NGS) data is vital for understanding the genetic basis of diseases and for targeted therapies [5]. In the realm of liquid biopsies utilizing cell-free DNA (cfDNA), detecting somatic copy number amplifications is increasingly recognized as an invaluable tool for early diagnosis [6], disease monitoring [7], and therapy selection, particularly for cancer patients [8].

Despite its clinical utility, CNV calling in cfDNA is difficult for reasons that are less acute in tumor tissue. cfDNA is highly fragmented [9] and exists in low concentrations [10], thereby necessitating specialized pre-processing steps [11] and higher sequencing depth to detect the weak signal. Existing CNV callers designed for bulk tumor tissue may not perform well under these conditions due to their assumptions about tumor purity and clonal heterogeneity.

A further complication is that many clinical workflows rely on targeted panels rather than whole-genome or whole-exome sequencing. These panels are cost-effective and focus on clinically relevant genes, but their narrow genomic scope can reduce the availability of control regions and make common model assumptions inappropriate [12]. Many CNV callers were developed for WGS/WES and are not optimized for targeted panel data, as we describe more in depth in the related work section [13]. As such, most methods fail to run or provide accurate calls in this setting. Furthermore, many callers do not function in small batch sizes commonly encountered in clinical settings. Given these constraints and limitations of existing CNV calling methods, there is a pressing need for a novel, optimized somatic CNV caller specifically designed for detecting copy number amplifications in cfDNA at low tumor content from targeted panels.

In this paper, we introduce BayesCNV, a state-of-the-art Bayesian somatic CNV caller tailored for these specific challenges. This model uses a Bayesian hierarchical model of the copy ratio compared to a normal sample, with gene-level means and variances to estimate the copy number. We derive this caller from basic assumptions and develop an inference scheme for estimating the parameters, making gene-specific calls, and calculating variant call confidence. We also develop a method, based on thermodynamic integration [14], for estimating the Bayesian evidence of the sample. This enables quality control and automatic detection of low-quality or mismatched samples. We then demonstrate the performance of this caller on data coming from Seracare^®^ samples (Milford USA) using two OncoReveal^®^ Liquid Biopsy Panels (Natick USA). These results are compared to two competitor methods, IonCopy [15] and DeviCNV [16], showing superior performance.

The contents of this paper are as follows: in Section 2 we report our literature review on current CNV callers and describe why most extant callers are unable to be used in our specific applications. In Section 3, we describe our development of the mathematical model with all steps required in inference, computation, and calling. In Section 4, we present the performance of our model on synthetic and real data. Finally, in Section 5 we provide some brief remarks on future directions for this work. An open-source implementation of the mathematical model is available at https://github.com/Pillar-Biosciences-Inc/BayesCNV (accessed on 1 December 2025).

## 2. Related Work

There is a large body of work on CNV detection from NGS data. However, among the methods we evaluated, only DeviCNV [16] and IonCopy met the practical requirements of targeted liquid biopsy panels in our setting. In particular, many publicly available CNV callers are not well-suited to clinical cfDNA workflows because they (1) rely on CNVs spanning long contiguous genomic regions, (2) require large cohorts for normalization or denoising, (3) do not explicitly estimate copy number magnitude, and/or (4) do not support calling both gains and losses. Table 1 summarizes the tools considered in our review.

Many CNV callers were developed for WES/WGS, such as cn.MOPS [17], CNVkit [18], and EXCAVATOR2 [19]. These methods are optimized for extremely low per-amplicon read depths, often below 100 reads. To mitigate high variance at individual targets, these methods often aggregate signal across longer contiguous regions, implicitly assuming that CNVs span large genomic intervals (typically >20 kb). While this improves stability by averaging out target-level noise, it can reduce sensitivity for short, focal CNVs that are common in clinical applications.

Another class of callers, such as Canoes, CoNIFER, and XHMM [20,21,22], relies on large cohorts of normal or pseudo-normal samples for denoising. For example, cn.MOPS [17] leverages reference normal samples to build an accurate baseline read-depth profile, while XHMM and CoNIFER apply a singular value decomposition to remove systematic variation across the samples. These requirements are often incompatible with clinical liquid biopsy workflows, which typically process small batches of samples in order to minimize turnaround time. Additional limitations across many CNV callers include an inability to detect both gains and losses, or to estimate the magnitude of the CNV (e.g., distinguishing between three and four copies).

The only three tools we identified that are explicitly designed for targeted panels are DeviCNV [16], IonCopy [15], and StateCNV [23]. StateCNV was designed for targeted panels with strong spatial correlation among amplicons and is therefore not well matched to liquid biopsy panels whose targets are distributed across the genome, as considered here. However, the other two are suitable competitors. DeviCNV employs a regression-based framework: it computes amplicon-level depth, normalizes it using sample-wide depth to account for GC content and PCR bias, and compares it against a null distribution under a diploid assumption. IonCopy uses a two-step normalization procedure, first across amplicons and then across samples, and constructs a robust null distribution to assign per-amplicon significance. Both methods were validated on targeted sequencing data and support CNV calling at high resolution, making them relevant baselines for our work.

**Table 1:** A Summary of Common Publicly Available CNV Callers.

Tool	PMID Reference	Year	High Spatial Res	Small Sample Support	Quantify CNV	Gain and Losses
Canoes [20]	24771342	2014	N	N	Y	Y
CLAMMS [24]	26382196	2015	Y	Y	N	Y
Cn.MOPS [17]	22302147	2012	N	N	Y	Y
CNVkit [18]	27100738	2016	N	Y	Y	Y
CODEX [25]	25618849	2015	N	Y	Y	Y
CoNIFER [21]	22585873	2012	N	N	Y	Y
DeviCNV [16]	30326846	2018	Y	Y	Y	Y
EXCAVATOR2 [19]	27507884	2016	N	Y	Y	Y
ExomeDepth [26]	22942019	2012	N	Y	Y	Y
ExonDel [27]	25322818	2014	Y	Y	Y	N
FishingCNV [28]	23539306	2013	N	N	Y	Y
HMZDelFinder [29]	27980096	2017	N	Y	Y	N
IonCopy [15]	26910888	2016	Y	Y	Y	Y
XHMM [22]	23040492	2012	N	N	Y	Y

## 3. Materials and Methods

### 3.1. Overall Processing Steps

**Figure 1:**
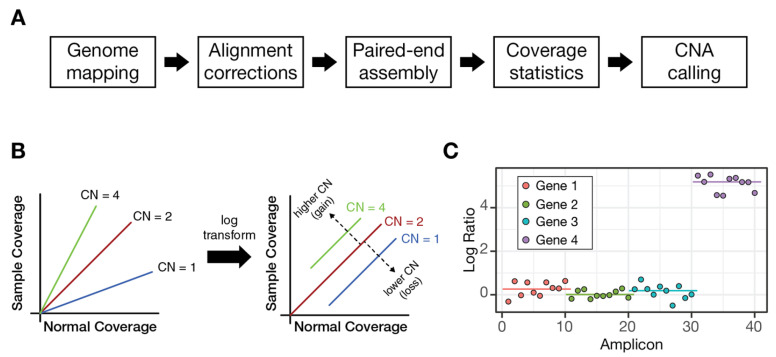
(**A**) The overall CNV pipeline. The reads are first mapped to the HG19 genome. This is followed by proprietary denoising steps and paired-end assembly. At this point, coverage statistics are computed (read depth), which are used by the caller described in this work. (**B**) An illustration of the primary assumption of the caller, the slope of reads in the sample compared to the normal depends on the number of copies. With a log transform this instead becomes a different intercept. (**C**) The different ratios of amplicons can be used to generate gene-level calls.

The PiVAT analysis pipeline begins with FASTQ files, which are aligned to the GRCh37/hg19 reference genome using the Burrows-Wheeler Aligner (BWA) [30] version bwa-0.7.16a. The resulting BAM files are processed with SAMtools [31] version 1.10 to compute alignment and coverage metrics, including per-target read counts and summary coverage statistics. Filtered and refined alignments are then used to derive amplicon-specific read counts, which serve as inputs for downstream CNV analysis. In subsequent steps, read counts are normalized and transformed to produce gene-level CNV estimates. A schematic overview of the pipeline is shown in Figure 1.

### 3.2. Biological Assumptions

Our CNV caller is based on a small set of biologically motivated assumptions. Specifically, we make the following assumptions:

1. Linearity of read counts. Expected read counts scale approximately linearly with copy number. For example, a locus present at four copies yields, on average, twice as many reads as a locus with two copies.
2. Sample-composition invariance. Observed read depth reflects the total DNA mixture in the sample, and tumor-derived DNA is processed similarly to background DNA within the assay. Consequently, the copy number scales with tumor fraction.
3. Stable amplicon-specific effects. While amplification efficiency varies across amplicons, these effects are assumed to be consistent across samples processed under comparable conditions. Therefore, the case and normal samples should be process-matched.
4. Sparsity of CNVs. The majority of the amplicons target loci are assumed to be copy-neutral, with CNVs affecting only a minority of targets.

These assumptions motivate the data transformations described as follows. Assumptions 1 and 2 relate the read depths to the copy number to be inferred. Namely, the relationship between copy number and read depth is linear (Assumption 1) and modeling of the tumor content is unnecessary (Assumption 2). We consider read counts from J targeted amplicons, obtained from a case sample and process-matched normal using the PiVAT pipeline. Let ${\left\{s_{j}\right\}}_{j=1}^{J}$ denote the read counts for the case sample and ${\left\{n_{j}\right\}}_{j=1}^{J}$ the corresponding counts from the process-matched normal (after filtering any amplicons that failed to amplify in either the sample or normal). To assess copy number variations, we compute the log copy ratio for each amplicon:

(1) $${\tilde{x}}_{j}=\log s_{j}-\log n_{j}=\log\frac{s_{j}}{n_{j}}$$

The normal sample accounts for amplicon-specific effects in read counts (Assumption 3). This reference may be explicitly specified by the user, or for normal-free calling, approximated by the mean profile across samples within a batch. To account for sample-specific effects such as variation in DNA input or sequencing depth, we median-center the log ratios:

(2) $$x_{j}={\tilde{x}}_{j}-median\left({\left\{{\tilde{x}}_{j}\right\}}_{j=1}^{J}\right)$$

assuming that most amplicons are copy-number neutral (Assumption 4). The resulting values ${\left\{x_{j}\right\}}_{j=1}^{J}$ are referred to as the log copy number ratios (lCNRs), which ideally reflect the log relative copy number between the case and control. Our objective is to estimate the copy numbers of G genes (e.g., the ten genes targeted in OncoReveal^®^ Core Lbx panel).

### 3.3. Mathematical Modeling

Each amplicon targeting a gene g can be treated as a noisy observation of the underlying gene-level lCNR. Let $x_{ig}$ denote the median-centered lCNR for amplicon $i\in \left\{1,\ldots ,I_{g}\right\}$ targeting gene g∈{1,…,G}. We model $x_{ig}$ as arising from a shared latent signal $\mu_{g}$ representing gene-level lCNR, plus biological and technical variability.

We estimate these quantities using a Bayesian hierarchical model. Each gene-level mean is drawn from a global distribution centered at $\mu_{0}$, allowing information to be shared across genes while permitting gene-specific deviations. Each gene is also assigned a noise scale $\tau_{g}^{2}$ to reflect differing levels of measurement uncertainty. The full generative model is:

(3) $$\begin{array}{c} \mu_{0}\sim N\left(0,\nu \right),\\ \sigma^{2}\sim InvGamma\left(\alpha_{\sigma },\beta_{\sigma }\right),\\ \tau_{0}^{2}\sim InvGamma\left(\alpha_{\tau_{0}},\beta_{\tau_{0}}\right),\\ \mu_{g}|\mu_{0},\sigma^{2}∼N\left(\mu_{0},\sigma^{2}\right),\\ z_{g}\sim InvGamma\left(\alpha_{\tau },\beta_{\tau }\right),\\ x_{ig}|\mu_{g},\tau_{0}^{2},z_{g}∼SoftLaplace\left(\mu_{g},\tau_{g}^{2}=\tau_{0}^{2}z_{g}^{2}\right) \end{array}$$

**Figure 2:**
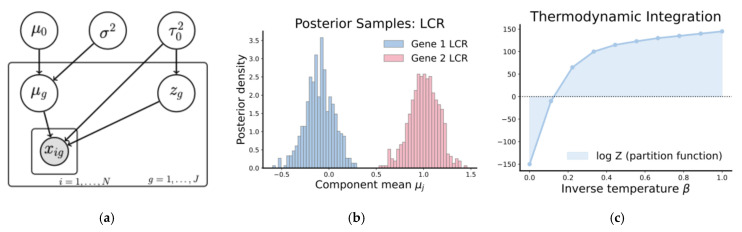
(**a**) The statistical model. The amplicon LCR is observed, colored in gray. Its distribution is parameterized by its mean, the global variance, and local variance. (**b**) A visualization of posterior samples for two gene LCRs derived from HMCMC. (**c**) Computation of the partition function via thermodynamic integration. The temperature is gradually decreased to the posterior, and the area under this curve corresponds to the log evidence of the statistical model.

The SoftLaplace distribution serves as a heavy-tailed alternative to the Gaussian, providing robustness to amplification outliers while remaining differentiable at the origin, which improves the numerical stability for gradient-based inference. Furthermore, the incorporation of a gene-specific noise term allows us to detect genes that have erratic empirical lCNRs as a quality control mechanism. A diagram of this model is shown on the left of Figure 2.

Intuitively, this hierarchical structure reflects our belief that most genes cluster around a common lCNR (e.g., diploid baseline) but may deviate due to biological variation or technical artifacts. By pooling information across amplicons within a gene, the model reduces noise and yields more stable estimates of $\mu_{g}$, especially when individual amplicons are noisy or unreliable [32]. At the same time, the per-gene variance $\tau_{g}^{2}$ accommodates genes with inherently noisier measurements. The SoftLaplace distribution provides robustness to outliers that are frequently observed in amplicon-based assays, such as extreme GC-content effects, while retaining smoothness necessary for gradient-based optimization.

### 3.4. Calling CNVs via Posterior Distribution

For convenience, we denote the set of model parameters by $\theta =\left\{\mu_{0},\sigma^{2},{\left\{\mu_{g}\right\}}_{g=1}^{G},\tau_{0}^{2},{\left\{z_{g}\right\}}_{1}^{G}\right\}$. Given observed amplicon-level lCNRs ${x=\left\{x_{j}\right\}}_{j=1}^{J}$, the posterior of our model is

(4) $$p\left(\theta |x\right)=\frac{p\left(\theta \right)p\left(x|\theta \right)}{\int p\left(\theta \right)p\left(x|\theta \right)d\theta }$$

where the joint prior and likelihood factorize according to the generative model:

(5) $$p\left(\theta \right)=p\left(\mu_{0}\right)p\left(\sigma^{2}\right)p\left(\tau_{0}^{2}\right)\prod_{g=1}^{G}p\left(\mu_{g}\right|\mu_{0},\sigma^{2})p(z_{g}),\;p\left(x|\theta \right)=\prod_{g=1}^{G}\prod_{i=1}^{I_{g}}p(x_{ig}|\mu_{g},\tau_{g}^{2}),\;\;\;\;\;\;\;\;\;\;\;\;\;\;\;\;\;\;\;\;\;\;\tau_{g}^{2}=\tau_{0}^{2}z_{g}^{2}$$

As stated previously, this model is designed to be easily interpretable: $\mu_{g}$ represents the gene-level lCNR for gene g. We therefore use the marginal posterior $p\left(\mu_{g}|x\right)$ to make CNV calls using two criteria:

1. Effect size threshold. The posterior mean should exceed a minimum magnitude, that is $E\left[p\left(\mu_{g}|{\left\{x\right\}}_{i=1:N}\right)\right]>\log T$, where T is the copy ratio we wish to be able to detect
2. Confidence threshold. The posterior probability close to 0 (CNV neutral) should be small: $\mathrm{Pr}\left(\left|\mu_{g}\right|<\delta \right)<\epsilon$, for some values of δ, ϵ. Here, δ indicates the size of variation we expect neutral genes to have and ϵ is the probability of false positive we are willing to accept.

Together, these criteria ensure that only sufficiently large and well-supported deviations from neutrality result in CNV calls.

### 3.5. Markov Chain Monte Carlo Inference

To perform inference over the hierarchical model, we use Markov chain Monte Carlo (MCMC) to draw samples from the posterior distribution [33]. In particular, we employ Hamiltonian Monte Carlo (HMC), a gradient-based MCMC algorithm that improves efficiency over traditional random-walk Metropolis approaches [34]. By introducing auxiliary momentum variables and simulating Hamiltonian dynamics, HMC can propose larger moves in the parameter space while often maintaining high acceptance rates. This results in faster mixing and more efficient exploration of complex, high-dimensional posteriors. Furthermore, we improved the geometry of the parameter space via reparameterization, a common trick in HMC to reduce curvature and improve mixing [34]. We found that MCMC on our dataset with 10 genes and 441 amplicons was very quick, roughly 10 s for a single clinical sample.

To eliminate the need to hand-tune trajectory lengths, we use the No-U-Turn Sampler (NUTS), a self-tuning extension of HMC [35]. NUTS adaptively builds a set of candidate proposals by simulating forward and backward along the Hamiltonian trajectory and terminates when a “U-turn” is detected, when the trajectory would return to a previously explored space. This dynamic trajectory length allows NUTS to balance computational cost with effective exploration automatically. A visualization of posterior samples is provided in the middle of Figure 2. We evaluated convergence using standard metrics for HMC, in particular ensuring that the acceptance probability was in a reasonable range (0.6–0.9), no divergent transitions (large differences in the starting and ending Hamiltonian), and effective sample size (a measure roughly quantifying the number of “independent” samples). We used 10,000 iterations, which is large, particularly for a relatively simple Bayesian model [32].

### 3.6. Quality Control via Likelihood Evaluation

Previous work has shown that the Bayesian model evidence (marginal likelihood) can serve as a useful diagnostic for detecting model-data mismatch and other pathologies [23]. The evidence is defined as

(6) Z=p(x)=∫p(x|θ)p(θ)dθ

Direct evaluation of Z is generally intractable for high-dimensional models. A common but unreliable approach is the harmonic mean estimator, which uses posterior samples $\theta^{\left(i\right)}∼p\left(\theta |x\right)$ to estimate Z via

(7) $${\hat{Z}}_{HME}={\left(\frac{1}{N}\sum_{i=1}^{N}\frac{1}{p\left(x|\theta^{\left(i\right)}\right)}\right)}^{-1}$$

The HME is well known to be unstable and can have infinite variance because it is dominated by rare samples in the low-likelihood tails. As such, it should be avoided in spite of its continued use [36].

More reliable approaches transform evidence computation into better-behaved expectations. Several alternatives exist including bridge sampling [14] and annealed importance sampling [37]. In this work, we use thermodynamic integration (TI) [14]. TI constructs a continuous path between the prior and posterior using a family of tempered (power posterior) distributions indexed by β:

(8) $$p_{\beta }\left(\theta |x\right)=\frac{p{\left(x|\theta \right)}^{\beta }p\left(\theta \right)}{Z(\beta )}$$

which interpolates between the prior (β=0) and posterior (β=1). TI exploits the identity

(9) $$\log Z=\int_{0}^{1}E_{\theta ∼p_{\beta }(\theta |x)}\left[\log p\left(x|\theta \right)\right]d\beta$$

expressing the log evidence as an integral over expectations under these tempered distributions. In practice, we approximate this integral by evaluating the expectation at a discrete set of temperatures $\{\beta_{k}{\}}_{k=1:K}$ and applying the trapezoidal rule

(10) $$\hat{\log Z}=\sum_{k=1}^{K-1}\frac{\beta_{k+1}-\beta_{k}}{2}\left({\hat{\mu }}_{\beta_{k}}-{\hat{\mu }}_{\beta_{k+1}}\right)$$

where

(11) $${\hat{\mu }}_{\beta_{k}}=\frac{1}{N}\sum_{i=1}^{N}\log p\left(x|\theta_{\beta_{k}}^{\left(i\right)}\right)$$

is the Monte Carlo estimate of the expectation of $p_{\beta_{k}}\left(\theta |x\right)\;$ using N MCMC samples ${\left\{\theta_{\beta_{k}}\right\}}_{i=1}^{N}$. This procedure is visualized on the right of Figure 2. This was more computationally intensive than the MCMC of Section 3.5, as we set K to 20, resulting in a runtime of roughly 2 min per clinical sample. We used the same diagnostics to ensure a convergent chain.

### 3.7. Code Availability

The model used in this paper is implemented in NumPyro version 0.11.0. This package allows for models to be implemented with a small amount of code while maintaining computational efficiency. This implementation is publicly available at https://github.com/Pillar-Biosciences-Inc/BayesCNV (accessed on 1 December 2025), along with demonstrations of how to use it on synthetic datasets.

## 4. Results

We evaluated the performance of BayesCNV using Seraseq^®^ ctDNA Complete Mutation Mix contrived samples that contain known ERBB2 and MET copy number amplifications. Samples were tested using the OncoReveal^®^ Core Lbx panel (441 amplicons targeting a variety of cancer-related mutations, including CNVs in 10 genes). We used a minimum call threshold of a minimal call threshold of 1.5, corresponding to three copies relative to a diploid baseline. We compared our method to IonCopy and DeviCNV. For IonCopy, we used gene-level calling, Bonferroni correction and global *p*-value adjustment. We left the *p*-value threshold at a default of 0.05 to compensate for the anticipated overly conservative nature of a Bonferroni correction. For DeviCNV, we set the user-controlled parameters of using the PCR data type, deduplicating reads, and setting a quality score threshold of 0. We also ensured the threshold matched our target of a 1.5 copy ratio.

### 4.1. Comparing BayesCNV to DeviCNV

We first compared BayesCNV with IonCopy and DeviCNV using 84 samples, a mixture of internal clinical normal and eight Seraseq Complete Mutation Mix CNV-positive samples at 5%, 2.5% and 1.25% VAF. Expected calls and copy numbers were determined via digital droplet PCR. One sample had sufficiently low concentration such that only the ERBB2 CNV was expected to be detected. IonCopy achieved the highest sensitivity at 0.93, with BayesCNV closely following at 0.87. In contrast, DeviCNV failed to identify any true positives, resulting in a sensitivity of 0. While IonCopy demonstrated marginally higher sensitivity, it did so at the cost of markedly reduced specificity. IonCopy produced 159 false positives, compared to 32 for DeviCNV and only 3 for our method, corresponding to specificities of 0.81, 0.96, and 0.996, respectively. Notably, by applying a conservative log-likelihood threshold of −300, corresponding to the bottom 5% of samples, we were able to eliminate all false positives from our method entirely. A summary of these results is presented in Table 2.

### 4.2. Limit of Detection with Synthetic Data

We next quantified the limit of detection (LOD) using semi-synthetic dilution experiments. Starting from a Seraseq-positive sample Section 4.1 with known ERBB2 and MET amplifications, we generated a series of in silico mixtures by scaling the ERBB2/MET amplicon counts to progressively weaker copy-number signals, down to an approximately diploid baseline. We then added noise calibrated to the empirical variance observed in CNV-neutral genes to mimic realistic assay variability.

**Figure 3:**
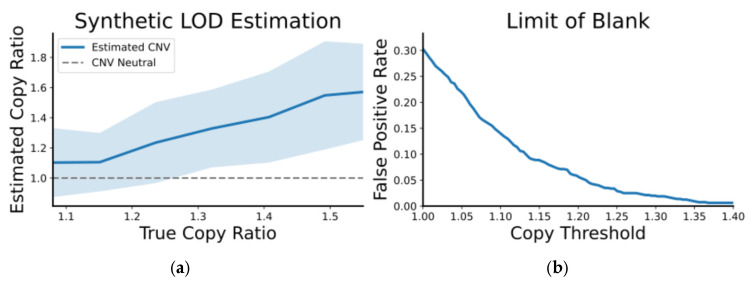
Limit of detection and false positive rate for CNV detection. Semi-synthetic datasets were generated by progressively down-scaling read counts from Seracare positive samples for the MET and ERBB2 loci and adding noise consistent with CNV-neutral variance. (**a**) The estimated CNV ratios from our caller as a function of the true copy number, with shaded regions indicating 90% confidence intervals. (**b**) The corresponding false positive rate as a function of the detection threshold. Reliable detection is achieved at a copy ratio of approximately 1.3, corresponding to 2.6 copies, with an associated false positive rate of about 2%.

We then analyzed each simulated dataset with BayesCNV and summarized the inferred copy ratio as a function of the ground-truth copy number. As shown in Figure 3 (left, 90% credible intervals), reliable CNV detection emerges at an approximate copy ratio of 1.3 (absolute copy number approximately 2.6). Using the same simulations, we estimated the false positive probability as a function of the calling threshold on the remaining CNV-neutral genes. The false-positive rate drops rapidly with increase ng threshold and is approximately 2% at a copy-ratio cutoff at 1.3.

### 4.3. Likelihood-Based Sample Filtering

As a final illustration of BayesCNV’s utility, we demonstrate how the TI estimate of the marginal log likelihood can serve as a discriminator of sample quality. Whereas Section 4.1 suggested that evidence-based quality control can reduce false positives, here we directly evaluate whether the log evidence separates known poor-quality samples from high-quality samples. For this experiment, we used a second dataset consisting of formalin-fixed paraffin-embedded (FFPE) samples processed with the OncoReveal^®^ Essential Lbx panel. Of the 32 samples, 10 were derived from highly degraded FFPE tissue. The DNA templates extracted from these samples amplify poorly in amplicon- or PCR-based assays due to damage sustained during the fixation process. The remaining 22 samples were high quality FFPE samples that were acquired from BioIVT.

**Figure 4:**
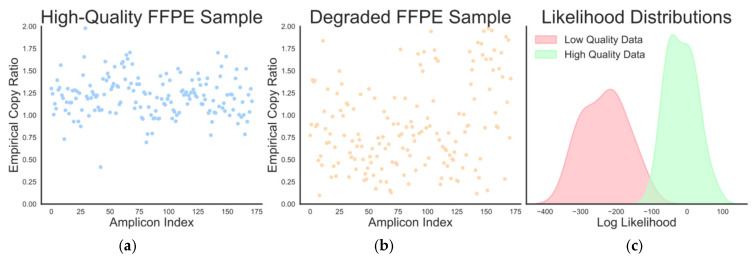
Likelihood-based quality control. (**a**) The empirical copy ratios of the high-quality sample. (**b**) The empirical copy ratios of a degraded sample. Notice the wider spread and erratic ratios. (**c**) The distributions of the likelihoods of the low- and high-quality data. A threshold of −100 would perfectly distinguish the two groups.

For each sample, we fit the hierarchical model on each of these samples using the cohort batch mean as the reference normal and computed the log-likelihood. Figure 4 (left and middle panels) plots the empirical copy ratio profile for a high-quality and degraded sample, respectively. Notably, traditional sequencing QC metrics such as read depth and on-target rate did not flag these samples as problematic.

Degraded samples exhibited markedly lower marginal log likelihoods, with an average of −232 ± 35. In contrast, the average log-likelihood was substantially higher at −15 ± 17. The worst observed log likelihood among the high-quality samples was −87, while the best (highest) degraded sample was only −137. As such, any threshold between these two values would provide perfect distinguishing ability between the two groups in this dataset.

In practice, a more conservative (lower) threshold may be preferable to account for limited training size and potential clinical variability. However, these data suggest that samples exhibiting dramatically lower likelihoods in the Essential panel (e.g., below −200) can be flagged for possible sample/normal mismatch or assay artifacts, and downstream CNV calls should be reviewed. Overall, these findings suggest that the marginal log-likelihood offers a principled, data-driven quality control metric in the BayesCNV workflow.

## 5. Discussion

In this study, we present a novel Bayesian framework for detecting and quantifying CNVs in targeted amplicon liquid biopsy data. The approach is grounded in fundamental biological and technically motivated assumptions about amplification behavior, resulting in a model that pools information across amplicons within each gene. Posterior inference is performed via Markov Chain Monte Carlo (MCMC), enabling posterior inference of gene-level copy numbers with uncertainty estimates, an important feature in clinical applications. We benchmarked our caller against two publicly available CNV detection methods. Our method demonstrated superior sensitivity and specificity, emphasizing its utility in clinical settings. Beyond CNV detection, we also introduce an evidence-based quality-control method based on the marginal log likelihood of the data. Estimating this quantity was performed via thermodynamic integration, a technique from statistical physics. On a second dataset consisting of FFPE-derived samples of varying quality, we showed that TI-derived log likelihoods clearly separated high-quality samples from degraded ones, even when traditional metrics did not distinguish them. This suggests that this quantity can serve as a powerful, model-based quality control metric in clinical applications.

There are several directions for improving BayesCNV. From a mathematical standpoint, integrating SNP-based tumor fraction estimation as a preprocessing step could help determine whether CNV calling is unlikely to be reliable, particularly in low-input settings. Eliminating samples where tumor content is too low to make viable calls could further reduce the false positive rate. Furthermore, while TI provides an accurate estimate of the likelihood, it is computationally intensive. Alternative estimators, such as annealed importance sampling, Rao-Blackwellized tempered sampling, and bridge sampling may offer more efficient approximations of the marginal likelihood, potentially reducing computation without compromising accuracy.

From a scientific standpoint, a natural direction is for single-cell applications. Recent studies have demonstrated that integrating bulk and single-cell omics data can substantially improve prediction of immunotherapy response by resolving tumor heterogeneity at the cellular level [38,39]. Several adaptations of our framework could enable such integration. First, the hierarchical structure of BayesCNV, which pools information across amplicons within genes, could be extended to pool information across cells within clonal populations, similar to how BayesPrism leverages single-cell references for Bayesian deconvolution of bulk RNA-seq data [40]. The SoftLaplace likelihood employed in BayesCNV is particularly well-suited to single-cell data, where amplification artifacts and dropout events produce heavy-tailed noise distributions [41]. Second, our thermodynamic integration-based quality control metric could be adapted to identify low-quality cells or detect outlier populations, addressing a persistent challenge in single-cell CNV analysis [42]. Third, single-cell CNV profiles could serve as reference signatures for deconvolving clonal substructure from bulk cfDNA, potentially enabling inference of clone-specific copy number states and their relative abundances within a liquid biopsy sample. This would be particularly valuable for tracking clonal evolution during therapy and identifying resistant subclones. Finally, following the paradigm established by the Scissor algorithm [43], single-cell-derived CNV signatures could be linked to clinical phenotypes such as immunotherapy response, enabling identification of CNV-defined cell populations that drive treatment outcomes. Such extensions would require addressing technical challenges inherent to single-cell CNV detection, including substantially lower per-cell coverage and the need to distinguish true biological heterogeneity from technical noise.

## Figures and Tables

**Table 2:** Performance on the OncoReveal^®^ Core Lbx Panel.

Method	TP	TN	FP	FN	Sens	Spec
IonCopy	14	686	159	1	0.93	0.81
DeviCNV	0	813	32	15	0	0.96
BayesCNV	13	842	3	2	0.87	0.996
BayesCNV + QC	13	825	0	2	0.87	1

## Data Availability

The raw data supporting the conclusions of this article will be made available by the authors on request.

## Abbreviations

The following abbreviations are used in this manuscript:

CNV	Copy Number Variation
FFPE	Formalin-Fixed Paraffin-Embedded
HMCMC	Hamiltonian Monte Carlo
lCNR	Log Copy Number Ratio
LOD	Limit of Detection
MCMC	Markov chain Monte Carlo
NGS	Next-Generation Sequencing
NUTS	No U-Turn Sampler
QC	Quality Control
TI	Thermodynamic Integration
VAF	Variant Allele Frequency
WES	Whole-Exome Sequencing
WGS	Whole-Genome Sequencing
